# Clinical efficacy of acupoint application therapy combined with Kangyou Decoction in treating *Helicobacter pylori* infection with damp-heat syndrome of the middle Jiao

**DOI:** 10.1186/s41065-025-00533-1

**Published:** 2025-08-14

**Authors:** Shang-Ren Xie, Meng-Yi Lv, Jun Yang, Hai-Jing Chen, Jie-Ru Wu, Yao-Qi Xu, Jie Jin, Jian-Wei Jin

**Affiliations:** 1 Department of Traditional Chinese Medicine, Wenzhou Central Hospital, Wenzhou, 325000 China; 2 Department of Gastroenterology, Wenzhou Central Hospital, No. 252 Baili East Road, Wuma Sub-District, Wenzhou, 325000 China

**Keywords:** Acupoint application, Bismuth quadruple therapy, Clinical efficacy, Damp-heat syndrome of the middle jiao, *Helicobacter pylori* infection, Kangyou decoction

## Abstract

**Background:**

This study evaluated the clinical efficacy and safety of acupoint application of traditional Chinese medicine (TCM) combined with Kangyou Decoction in patients with *Helicobacter pylori* (*Hp*) infection presenting with damp-heat syndrome obstructing the middle jiao.

**Methods:**

A total of 416 patients with *Hp* infection and TCM-defined damp-heat syndrome of the middle jiao were enrolled and randomly assigned to a treatment group (*n* = 208) or a control group (*n* = 208). The control group received standard bismuth-containing quadruple therapy, while the treatment group received acupoint application of TCM combined with oral administration of Kangyou Decoction. Both groups underwent treatment for two weeks. Outcomes included Hp eradication rates, changes in TCM syndrome scores pre- and post-treatment, and the incidence of adverse reactions.

**Results:**

Following two weeks of therapy, no statistically significant difference in *Hp* eradication rates was observed between the two groups (*p* > 0.05). Both groups demonstrated significant improvement in TCM syndrome scores (*p* < 0.05), with the treatment group showing a greater reduction compared to the control group (*p* < 0.05). The total effective rate in the treatment group was higher than that in the control group. Incidences of adverse reactions did not differ significantly between the groups (*p* > 0.05).

**Conclusion:**

Acupoint application combined with Kangyou Decoction yielded comparable Hp eradication outcomes to bismuth quadruple therapy in patients with damp-heat syndrome of the middle jiao. The combined therapy demonstrated greater improvement in clinical symptoms with a favorable safety profile, supporting its potential for clinical application.

## Background

*Helicobacter pylori* (*Hp*) is a gram-negative bacterium with a high global prevalence and is closely associated with the pathogenesis of several chronic gastrointestinal diseases, particularly gastric adenocarcinoma and mucosa-associated lymphoid tissue (MALT) lymphoma, thereby constituting a significant global public health concern [1, 2]. In China, the infection rate remains substantial, with prevalence reported at 58.6% and 61.1% among patients with gastric ulcer and duodenal ulcer, respectively [3]. Although quadruple therapy containing bismuth is recommended as the first-line strategy for both initial and subsequent eradication attempts, its clinical efficacy is often limited by factors such as antimicrobial resistance and adverse drug reactions, resulting in suboptimal eradication rates [4, 5].

Within the framework of traditional Chinese medicine (TCM), symptoms associated with *Hp* infection are typically classified under diagnostic categories such as “stomach stuffiness disease” (Wei Pi) or “epigastric pain” (Wei Tong). Treatment is guided by the principle of syndrome differentiation. Previous research has indicated a higher susceptibility to *Hp* infection among patients with a damp-heat constitution, underscoring the therapeutic importance of interventions aimed at clearing heat and resolving dampness in this population [6, 7]. 

Based on these considerations, a total of 416 patients with TCM-defined damp-heat syndrome and confirmed *Hp* infection, who received care at the Wenzhou Central Hospital between January and October 2024, were enrolled in the current study. The therapeutic outcomes of a TCM-based treatment protocol were assessed and are presented below.

## Materials and methods

### General information

This study was conducted between January and October 2024 across the Department of Gastroenterology, Physical Examination Center, and Department of Traditional Chinese Medicine at Wenzhou Central Hospital. A total of 416 patients with *Hp* infection and TCM-defined damp-heat syndrome were enrolled following the provision of written informed consent. Participants were randomly assigned to either the treatment or control group in a 1:1 ratio.

The control group included 208 participants (105 males and 103 females), aged 20 to 68 years, with an average age of 46.93 ± 7.06 years and a disease duration ranging from 4 months to 3 years. This group received bismuth-containing quadruple therapy. The treatment group also included 208 participants (106 males and 102 females), aged 21 to 67 years, with an average age of 47.92 ± 7.12 years and a disease duration ranging from 3 months to 2 years. This group received a combination therapy consisting of TCM acupoint application and Kangyou Decoction.

This study followed the Declaration of Helsinki and was approved by the Ethics Committee of Wenzhou Central Hospital (2023-052). Written informed consent was obtained from all participants.

### Blinding and quality control

Stratified block randomization was utilized in this study, with stratification based on age (< 40 years or ≥ 40 years) and duration of *Hp* infection (≤ 1 year or > 1 year). The randomization sequence was generated using SAS version 9.4 with a seed number of 20,230,215. Allocation concealment was maintained through the use of sealed, opaque envelopes, which were prepared and managed by an independent third party—the Department of Science and Education at Wenzhou Central Hospital.

A single-blind design was adopted, in which outcome evaluators were blinded to group allocation. To ensure procedural consistency and minimize operator-related variability, all tasks related to the preparation of the TCM paste—including grinding and formulation—were conducted by physicians from the Department of Traditional Chinese Medicine who had undergone standardized training in relevant procedures. Application of the acupoint pastes was carried out by research nurses with substantial clinical experience to ensure adherence to protocol and maintain treatment consistency.

### Diagnostic criteria

The diagnostic criteria applied in this study were based on both Western medicine standards for *Hp* infection and TCM diagnostic guidelines for damp-heat syndrome.

① The diagnosis of *Hp* infection was established according to the *6th National Consensus Report on the Management of Helicobacter pylori Infection*, utilizing the carbon-13 urea breath test as the diagnostic method [8].
② The diagnostic criteria for damp-heat syndrome in patients with chronic gastritis were formulated in accordance with the *Expert Consensus on Traditional Chinese and Western Medicine Diagnosis and Treatment of Chronic Gastritis (2023).* [9] Required clinical manifestations included the presence of primary symptoms such as epigastric fullness or pain, generalized fatigue, and a sensation of heaviness, and sticky or loose stools. At least two secondary symptoms were additionally required, which included diminished or poor appetite, bitter taste in the mouth, bad breath, and mental fatigue. Tongue and pulse characteristics supportive of the diagnosis included a red tongue with a thick, greasy yellow coating and a slippery or rapid pulse. The diagnosis of damp-heat syndrome was established based on the presence of all primary symptoms, at least two secondary symptoms, and corroborative tongue and pulse findings.

### Inclusion criteria

Participants were eligible for inclusion if the diagnosis of a primary *Hp* infection was confirmed and no prior treatment had been administered. Eligibility also required fulfillment of the diagnostic criteria for damp-heat syndrome obstructing the middle jiao based on TCM principles. Additional inclusion criteria included an age range of 18 to 70 years, no use of antibiotics within the preceding month, and no use of acid inhibitors or gastric mucosal protectants within the past two weeks. Informed consent was voluntarily provided by all participants.

### Exclusion criteria

Exclusion criteria encompassed individuals with severe disorders of the gastrointestinal tract or other organ systems, impaired hepatic or renal function, current pregnancy or lactation, or a diagnosis of psychiatric illness. Also excluded were patients who had recently participated in other clinical studies or who exhibited known contraindications to the pharmacologic agents used in the present study.

## Treatment methods

### Control group

Participants in the control group received a bismuth-containing quadruple regimen. The treatment protocol included the following medications:

Amoxicillin Capsules (produced by CSPC ZhongNuo Pharmaceutical [Shijiazhuang] CO., Ltd.; National Medical Products Administration [NMPA] Approval Number: H13023964; Specification: 0.25 g): Administered orally at a dosage of 1000 mg twice daily, 30 min after breakfast and dinner.

Clarithromycin Sustained-release Tablets (manufacturer: Jiangsu Hengrui Medicine Co., Ltd.; NMPA Approval No: H20031041; Specification: 0.5 g): Administered orally at a dosage of 0.5 g twice daily, 30 min after breakfast and dinner.

Rabeprazole Sodium Enteric-coated Tablets (manufacturer: Weicai [China] Pharmaceutical Co., Ltd.; NMPA Approval Number: H20090091; Specification: 10 mg): Administered orally at a dosage of 10 mg twice daily, 30 min before breakfast and dinner.

Colloidal Bismuth Pectin Capsules (manufacturer: Shanxi Zhendong Anxin Biopharmaceutical Co., Ltd.; NMPA Approval Number: H20058476; Specification: 100 mg): Administered orally at a dosage of 0.2 g twice daily, 30 min before breakfast and dinner.

The duration of the treatment was 14 consecutive days.

### Treatment group

Participants in the treatment group received a combined intervention comprising TCM acupoint application and oral administration of Kangyou Decoction.

#### TCM acupoint application

The preparation for acupoint application was formulated using Rhizoma *Coptidis* (batch number: SC2023012, berberine content ≥ 5.2%) and *Magnolia officinalis* (batch number: SC2023009, magnolol content ≥ 2.1%) in a 1:1 ratio. The herbs were ground into fine powder and passed through a 200-mesh sieve. A total of 21 g of this powdered mixture was blended with 14 mL of ginger juice (pH 6.5–7.0, sourced from Leqing, Zhejiang) and 7 mL of honey (Baihua brand, Baume ≥ 42°) to form a homogeneous paste. This paste was divided into seven equal portions and molded into drug cakes (diameter 10.0 ± 0.5 mm, thickness 2.0 ± 0.2 mm).

The drug cakes were applied to the Zhongwan, bilateral Pishu, Weishu, and Zusanli (ST 36). Each application lasted for six hours and was conducted once daily over a 14-day treatment course.

**Kangyou Decoction**: The Kangyou Decoction consisted of the following herbal ingredients: Rhizoma *Coptidis* 9 g, *Magnolia officinalis* 10 g, Rhizoma *Acori tatarinowii* 9 g, Rhizoma *Pinelliae* preparata 12 g, Fructus *Gardeniae* preparata 9 g, Rhizoma *Phragmitis* 10 g, Herba *Taraxaci* 15 g, Radix *Glycyrrhizae* preparata 9 g, Radix *Salviae miltiorrhizae* 20 g, Radix *Notoginseng* 6 g, ark shell 30 g, and os sepiae 30 g. All medicinal materials were from a single batch (batch number: WZCHM202212) and complied with standardized quality specifications.

Decoction took place at the Traditional Chinese Medicine Pharmacy of Wenzhou Central Hospital using an automatic decoction machine. The herbs were initially soaked for 30 min. The first decoction involved the addition of 10 times the volume of purified water and boiling for 30 min. The second decoction used 8 times the volume of purified water and boiling for 20 min. The resulting filtrates from both extractions were combined and concentrated. The preparation process adhered to the quality control standards outlined in the 2020 edition of the *Pharmacopoeia of the People’s Republic of China*.

One dose of Kangyou Decoction was administered daily, to be taken warm in the morning and evening. The duration of treatment was 14 days.

### Observation indicators

#### Eradication rate of Hp

At four weeks following the completion of treatment, participants in both groups underwent a carbon-13 urea breath test using a 13 C infrared spectrometer (Anhui Yanghe Medical Device Co., Ltd.). A negative result was interpreted as successful eradication of Hp.

#### TCM syndrome score and evaluation of therapeutic efficacy

Assessment of TCM symptomatology was conducted based on predefined criteria: Primary symptoms included epigastric or abdominal distension or pain, generalized fatigue, and sticky or loose stools. Severity was scored as follows: 0 for none, 2 for mild, 4 for moderate, 6 for severe.

Secondary symptoms included decreased appetite, belching, heartburn, irritability, epigastric burning sensation, dry and bitter taste in the mouth, halitosis, sticky oral sensation, yellow urine, burning sensation around the anus, red tongue, yellow and greasy tongue coating, and slippery or rapid pulse. Scoring was as follows: 0 for none, 1 for mild, 2 for moderate, 3 for severe.

Therapeutic efficacy based on TCM syndrome scores was classified as:

##### Clinical cure

Disappearance or near-complete resolution of clinical symptoms and signs, with a reduction in total syndrome score of 95% or more.

##### Marked efficacy

Notable alleviation of clinical symptoms and signs, with a reduction in the score of 70% or more.

##### Effective

General improvement in symptoms and signs, with a reduction in the score of 30% or more.

##### Ineffective

Minimal or no significant improvement, or symptom worsening, with a reduction in the score of less than 30% (calculated based on the total syndrome score).

③ **Adverse events**: The occurrence of adverse reactions was documented throughout the study period.

### Data processing method

Statistical analyses were performed using SPSS software version 25.0. Quantitative data were presented as mean ± standard deviation (x ± s). Independent-samples t-tests were applied for comparisons between groups, while paired t-tests were used for within-group comparisons. Categorical data were expressed as percentages (%), with inter-group differences assessed using the chi-square test. A *p*-value less than 0.05 was considered statistically significant.

## Results

### Comparison of baseline characteristics between the two groups

No statistically significant differences were observed between the two groups with respect to age, gender, or disease duration (*p* > 0.05), indicating comparability in baseline characteristics (Table 1).

**Table 1:** Comparison of baseline characteristics between the two groups

			Gender		
**Group**	**n**	**Age**	**Male**	**Female**	**Disease duration (months)**
Treatment group	208	47.92 ± 7.12	106(51.0%)	102(49.0%)	11.96 ± 6.13
Control group	208	46.93 ± 7.06	105(55.5%)	103(49.5%)	12.45 ± 9.20
t		1.424	0.010		0.639
p		0.155	0.922		0.523

### Comparison of Hp eradication rates between the two groups

After 4 weeks of treatment, the eradication rate of *Hp* was 78.8% (164/208) in the treatment group and 84.1% (175/208) in the control group. The difference in eradication rates between the two groups was not statistically significant (χ² = 1.928, *p* = 0.165, Table 2).

**Table 2:** Comparison of *Helicobacter pylori* (*Hp*) eradication rates (n (%))

Group	Hp Eradicated	Hp Not Eradicated
Treatment group	164(78.8)	44(21.2)
Control group	175(84.1)	33(15.9)
x^2^	1.928	
*p* value	0.165	

Subgroup analysis indicated that participants in the treatment group with a disease duration of ≤ 1 year had an eradication rate of 88.0% (110/125), which was significantly higher than the 65.1% (54/83) observed in those with a disease duration > 1 year (χ² = 15.737, *p* = 0.000, Table 3).

**Table 3:** Subgroup analysis of *Helicobacter pylori* (*Hp*) eradication rates (n (%))

Subgroup	*n*	Hp Eradicated	Hp Not Eradicated
Disease duration of ≤ 1 year	125	110(88.0%)	15(12.0%)
disease Duration > 1 year	83	54(65.1%)	29(34.9%)
*x* ^*2*^		15.737	
*p*		0.000	

### Comparison of changes in TCM syndrome scores and treatment efficacy

Post-treatment assessments indicated a significant reduction in TCM syndrome scores in the treatment group, from 43.13 ± 2.99 points to 13.28 ± 11.36 points (*t* = 38.078, *p* < 0.001). In comparison, the control group exhibited a reduction from 42.97 ± 2.52 points to 22.03 ± 13.12 points (*t* = 23.010, *p* < 0.001). The comparison of the difference between the groups showed a significantly greater mean reduction in the treatment group by 8.91 points (95% CI: 6.55–11.27, *p* < 0.001), as shown in Table 4.

**Table 4:** Comparison of changes in primary TCM syndrome scores and total syndrome scores (points, x ± s)

	Abdominal Distension or Pain	Heavy Physical Fatigue	Sticky or Loose Stool	Main TCM Syndrome Scores	Total TCM Syndrome Scores
Treatment group before treatment	5.46 ± 0.89	5.64 ± 0.77	5.57 ± 0.83	16.67 ± 1.54	43.13 ± 2.99
Treatment group after treatment	1.65 ± 1.76	1.75 ± 1.72	1.7 ± 1.79	5.11 ± 4.61	13.28 ± 11.36
P^Δ^	0.000	0.000	0.000	0.000	0.000
Treatment group before treatment	5.38 ± 0.93	5.63 ± 0.78	5.57 ± 0.83	16.58 ± 1.59	42.97 ± 2.52
Treatment group after treatment	2.7 ± 2	2.85 ± 1.98	2.8 ± 1.87	8.35 ± 5.08	22.03 ± 13.12
P^Δ^	0.000	0.000	0.000	0.000	0.000
P^*^	0.000	0.000	0.000	0.000	0.000

Note: P^Δ^ represents comparison with the treatment group before the treatment; P* represents comparison between treatment and control groups post-intervention

The total effective rate, based on TCM efficacy evaluation criteria, was 91.8% (191/208) in the treatment group, which was significantly higher than the 67.9% (145/208) observed in the control group (χ² = 51.350, *p* = 0.000), as presented in Table 5.

**Table 5:** Comparison of therapeutic effects (n, %)

Group	Recovered	Significant Effect	Effective	Ineffective	Total Efficiency Rate	x^2^	*p* value
Treatment group	44	86	61	17	91.8%	51.350	0.000
Control group	18	47	80	63	67.9%		

### Comparison of adverse event incidence between the two groups

The incidence of adverse events was 1.4% (3/208) in the treatment group and 4.3% (9/208) in the control group, with no statistically significant difference between the groups (χ² = 3.119, *p* = 0.077). In the treatment group, all adverse events involved diarrhea (1.4%, 3/208), and none of the affected participants discontinued treatment. In the control group, adverse events included nausea (1.0%, 2/208) and diarrhea (1.4%, 3/208), with no treatment discontinuation observed in these cases. However, one participant discontinued treatment due to the occurrence of a rash, and three participants discontinued due to abnormal liver function. (Tables 6 and 7)

**Table 6:** Comparison of adverse reactions (Example (%))

Group	*n*	Adverse reactions			
		Nausea	Diarrhea	Rash	Abnormal Liver Function
Treatment group	208	0(0.0%)	3(1.4%)	0(0.0%)	0(0.0%)
Control group	208	2(1.0%)	3(1.5%)	1(0.5%)	3(1.4%)
X^2^		0.502	0.000	0.000	1.343
P		0.478	1.000	1.000	0.247

**Table 7:** Comparison of liver function parameters before and after treatment (mean ± SD)

Liver Function Indicators	*n*	Control group	Treatment group	t/z	*p*
Aspartate aminotransferase					
Before treatment	208	28.25 ± 3.58	27.84 ± 3.59	1.368	0.171
After treatment	208	36.21 ± 5.21	33.13 ± 4.92	5.887	0.000
Alanine aminotransferase					
Before treatment	208	25.46 ± 3.4	25 ± 3.01	1.320	0.187
After treatment	208	31.09 ± 3.35	29.18 ± 3.99	4.758	0.000
Total bilirubin					
Before treatment	208	10.42 ± 2.44	10.55 ± 2.49	0.553	0.581
After treatment	208	10.41 ± 2.58	10.51 ± 2.51	0.425	0.671

Note: Aspartate aminotransferase/alanine aminotransferase (U/L), total bilirubin (ummol/L)

## Discussion

*Hp*, a bacterium with a high global infection rate, has been recognized as a major contributor to the development of various chronic gastrointestinal and systemic diseases. Its persistent prevalence presents a substantial challenge to global public health initiatives [10]. 

In China, decades of clinical experience have led to the establishment of a diagnostic and therapeutic framework with distinct national characteristics for *Hp* infection management. The 2022 edition of the *Chinese Guideline for the Treatment of Helicobacter pylori Infection*, recommends the bismuth-containing quadruple regimen as the first-line approach for both initial and second-line eradication therapy, with a standard treatment duration of 14 days [11]. 

The guideline also highlights the need for flexibility in treatment strategies. In regions where the efficacy of the bismuth quadruple regimen is suboptimal, the use of empirical traditional Chinese medicine may be considered as an adjunct or alternative. For patients with refractory *Hp* infection, hypersensitivity to components of the bismuth quadruple regimen, or intolerance due to adverse reactions, TCM-based therapeutic modalities offer a viable alternative aimed at achieving eradication of the infection while minimizing treatment-related complications.

In TCM, *Hp* is conceptually classified as a “formless worm.” This notion is grounded in classical medical texts such as the *Huang Di Nei Jing*, which states: “Failing to regulate one’s diet can result in harm in the morning and damage in the evening, leading to a buildup that turns into accumulation, and after a long time, transforms into heat. This heat then generates dampness, and together, they give rise to all sorts of strange worms.” In the context of *Hp*-related symptoms such as epigastric distension and pain, TCM attributes the pathogenesis to disease patterns historically described as “distension,” “stomach ache,” and “damp-heat.”

The *Treatise on Damp-Heat Diseases* describes clinical manifestations such as “heaviness, epigastric stuffiness, and loose stools,” which are consistent with the syndrome of damp-heat accumulation observed in patients with *Hp* infection. According to the theoretical system of TCM, indiscretions—particularly excessive intake of rich, sweet, or greasy foods—are primary contributors to the development of damp-heat in the middle energizer (zhongjiao). This pathogenic damp-heat is believed to impair the regulatory functions of the spleen and stomach by obstructing the ascending movement of spleen qi and the descending movement of stomach qi.

Qing Dynasty physician Xue Shengbai provided further elaboration on the pathophysiology of damp-heat, stating: “The Yang heat of heaven and the Yin dampness of the earth interact to cause harm; when they unite, the heat intensifies and the dampness worsens. If dampness and heat separate, the condition remains mild and superficial; however if they remain together, the disease becomes acute and severe.” This description of pathogenesis, resembling the dense and adhesive nature of blended oil and flour, parallels the chronic and recurrent clinical course of *Hp* infection. Contemporary biomedical research supports the view that a damp-heat internal environment may facilitate *Hp* colonization and persistence. Such an environment not only promotes bacterial proliferation but also aggravates mucosal damage through complex inflammatory and immunological mechanisms, thereby perpetuating a pathogenic cycle. Although classical TCM literature lacks direct references to *Hp* as a microbial pathogen, extensive clinical experience in regulating spleen and stomach function, tonifying healthy qi, and eliminating pathogenic factors offers a complementary therapeutic framework for managing *Hp* infection using TCM [12, 13]. 

Acupuncture point application therapy using Chinese medicinal herbs represents a frequently utilized external modality in TCM. This method involves the direct application of herbal formulations to specific acupoints, with the aim of stimulating meridians and promoting the regulation of Qi and blood circulation. In the present study, the herbs Rhizoma *Coptidis* and *Magnolia officinalis* were selected based on their traditional functions, which include clearing heat, drying dampness, promoting Qi flow, and facilitating digestion.

The Zhongwan (CV 12) acupoint was selected to strengthen the spleen, harmonize the stomach, alleviate rebellious Qi, and promote diuresis. The Pishu (BL 20) point was used for strengthening the spleen, transforming dampness, and harmonizing the stomach and middle. The Weishu (BL 21) point was chosen for its function in harmonizing gastric activity, reducing reversed Qi flow, and regulating both Qi and blood. The Zusanli (ST 36) point was utilized due to its broad-spectrum effects, including spleen and stomach harmonization, reinforcement of vital energy, activation of channels and collaterals, and regulation of Qi dynamics.

Evidence from recent studies has demonstrated that stimulation of Zhongwan and Zusanli (ST 36) significantly increased serum IL-10 levels (*p* < 0.01) and decreased TNF-α (*p* < 0.05). These effects have been associated with modulation of the vagus nerve-immune regulatory pathway. The therapeutic mechanism of this treatment method involves not only the pharmacological action of the applied herbal substances but also the physiological stimulation of acupoints, which collectively enhance the circulation of Qi and blood along the meridians and visceral functions. This dual-action approach thus achieves both pharmacological and neurophysiological therapeutic effects.

The “Kangyou Decoction” used in this study was a modified formulation derived from the classical preparation “Coptis and Officinal Magnolia Bark Beverage.“ [14] Among its ingredients, Rhizoma *Coptidis* clears heat and dries dampness, cools the interior, and relieves stagnation; *Magnolia officinalis* promotes the flow of Qi, transforms dampness, and relieves stagnation; Rhizoma *Acori tatarinowii* transforms dampness and harmonizes the stomach; Rhizoma *Pinelliae* preparata reduces inverted flow and stops vomiting; Fructus *Gardeniae* preparata clears heat and promotes diuresis; Rhizoma *Phragmitis* generates moisture to relieve restlessness; Radix *Salviae miltiorrhizae* and Radix *Notoginseng* remove blood stasis and promote circulation; ark shell and os sepiae neutralize acid and harmonize the stomach; Herba *Taraxaci* clears heat and detoxifies; and Radix *Glycyrrhizae* preparata strengthens the spleen and benefits the stomach. The formulation aimed to address the pathophysiological features of damp-heat accumulation associated with *Hp* infection [15]. 

Modern pharmacological studies have demonstrated that berberine, an active alkaloid derived from *Coptis chinensis*, integrates into the lipid bilayer of *Hp* cell membranes, resulting in increased membrane permeability [16]. This alteration facilitates the efflux of intracellular ions, such as potassium (K⁺), and calcium (Ca²⁺), and subsequently inhibits adenosine triphosphate (ATP) synthesis. Additionally, berberine was found to directly kill *Hp* by inhibiting the activity of urease (IC₅₀ = 8.2 µM) and also regulate the intestinal flora by increasing the proportion of beneficial microorganisms such as *Bifidobacteria* and *Lactobacilli*, to repair the mucosal barrier [17]. 

Magnolol, a bioactive constituent of *Magnolia officinalis*, was observed to integrate into the *Hp* cell membrane through hydrophobic interactions, disrupting the arrangement of membrane lipids. This disruption enhanced membrane permeability and led to the extrusion of intracellular ions, such as K⁺, magnesium (Mg²⁺), and ATP. In addition, magnolol inhibited the expression of *Hp* virulence factors, such as CagA and VacA, ultimately leading to bacterial death [18]. 

Other herbal components, including Rhizoma *Acori tatarinowii*, Rhizoma *Pinelliae* preparata, Fructus *Gardeniae* preparata, Rhizoma *Phragmitis*, and Herba *Taraxaci*, exhibited varying degrees of antibacterial and anti-inflammatory activity. ark shell and os sepiae were reported to exert gastroprotective effects by inhibiting gastric acid secretion and neutralizing gastric acid, thus protecting the gastric mucosa. Radix *Notoginseng* and Radix *Salviae miltiorrhizae* contributed to improved gastric mucosal microcirculation, creating a more favorable environment for mucosal cell viability and enhancing resistance to *Hp*.

This study compared the therapeutic efficacy of TCM protocols and Western pharmacologic treatments in patients with *Hp* infection characterized by damp-heat obstruction. No statistically significant difference was observed in the Hp eradication rate between the TCM treatment group and the Western medicine treatment group (*p* > 0.05). However, a greater reduction in TCM syndrome scores was recorded in the TCM group, and the overall efficacy based on TCM clinical evaluation criteria was superior to that of the control group.

In addition, the use of acupoint application with TCM and the “Kangyou Decoction” was associated with fewer adverse drug reactions and a higher level of safety. These findings may be attributed to the natural origin of TCM constituents, which are generally characterized by low toxicity, mild side effects, and systemic regulatory properties, facilitating broader clinical applicability and acceptance.

In the treatment group, the significantly higher eradication rate in patients with a disease duration of ≤ 1 year than in those with a disease duration > 1 year (χ² = 15.737, *p* = 0.000). This finding suggests that shorter durations of *Hp* infection are associated with significantly improved eradication outcomes.

## Conclusion

In conclusion, the combined application of TCM acupoint application therapy and the “Kangyou Decoction” in the treatment of *Hp* infection characterized by damp-heat obstruction did not demonstrate superiority in eradication rates when compared with Western medicine treatments. However, this integrative approach was associated with significant clinical efficacy and a low incidence of adverse drug reactions, indicating a favorable safety profile. These findings suggest that this treatment modality may serve as an alternative therapeutic option for patients with damp-heat obstructive *Hp* infection who are unable to tolerate conventional pharmacologic regimens. Future studies with larger sample sizes and extended follow-up durations are warranted to further evaluate the long-term effectiveness and safety of this approach, thereby contributing to a more comprehensive evidence base for the clinical management of *Hp* infection.

## Data Availability

Data related to the current study are available from the corresponding author on reasonable request.

## Abbreviations

HP: Helicobacter pylori
ATP: Adenosine Triphosphate
IL-10: Interleukin – 10
TNF-α: Tumor Necrosis Factor – alpha
